# Genome-wide association mapping of quantitative traits in a breeding population of sugarcane

**DOI:** 10.1186/s12870-016-0829-x

**Published:** 2016-06-24

**Authors:** Josefina Racedo, Lucía Gutiérrez, María Francisca Perera, Santiago Ostengo, Esteban Mariano Pardo, María Inés Cuenya, Bjorn Welin, Atilio Pedro Castagnaro

**Affiliations:** Estación Experimental Agroindustrial Obispo Colombres (EEAOC)- Consejo Nacional de Investigaciones Científicas y Técnicas (CONICET), Instituto de Tecnología Agroindustrial del Noroeste Argentino (ITANOA), Av. William Cross 3150, Las Talitas, T4101XAC Tucumán Argentina; Departamento de Biometría, Estadística y Cómputos, Facultad de Agronomía, Universidad de la República, Garzón 780, 12900 Montevideo, Uruguay; Agronomy Department, University of Wisconsin – Madison, 1575 Linden Dr., Madison, WI 53706 USA

**Keywords:** Biomass, Linkage disequilibrium, Population structure, Quantitative trait loci (QTL), *Saccharum sp*, Sugar

## Abstract

**Background:**

Molecular markers associated with relevant agronomic traits could significantly reduce the time and cost involved in developing new sugarcane varieties. Previous sugarcane genome-wide association analyses (GWAS) have found few molecular markers associated with relevant traits at plant-cane stage. The aim of this study was to establish an appropriate GWAS to find molecular markers associated with yield related traits consistent across harvesting seasons in a breeding population. Sugarcane clones were genotyped with DArT (Diversity Array Technology) and TRAP (Target Region Amplified Polymorphism) markers, and evaluated for cane yield (CY) and sugar content (SC) at two locations during three successive crop cycles. GWAS mapping was applied within a novel mixed-model framework accounting for population structure with Principal Component Analysis scores as random component.

**Results:**

A total of 43 markers significantly associated with CY in plant-cane, 42 in first ratoon, and 41 in second ratoon were detected. Out of these markers, 20 were associated with CY in 2 years. Additionally, 38 significant associations for SC were detected in plant-cane, 34 in first ratoon, and 47 in second ratoon. For SC, one marker-trait association was found significant for the 3 years of the study, while twelve markers presented association for 2 years. In the multi-QTL model several markers with large allelic substitution effect were found. Sequences of four DArT markers showed high similitude and e-value with coding sequences of *Sorghum bicolor*, confirming the high gene microlinearity between sorghum and sugarcane.

**Conclusions:**

In contrast with other sugarcane GWAS studies reported earlier, the novel methodology to analyze multi-QTLs through successive crop cycles used in the present study allowed us to find several markers associated with relevant traits. Combining existing phenotypic trial data and genotypic DArT and TRAP marker characterizations within a GWAS approach including population structure as random covariates may prove to be highly successful. Moreover, sequences of DArT marker associated with the traits of interest were aligned in chromosomal regions where sorghum QTLs has previously been reported. This approach could be a valuable tool to assist the improvement of sugarcane and better supply sugarcane demand that has been projected for the upcoming decades.

**Electronic supplementary material:**

The online version of this article (doi:10.1186/s12870-016-0829-x) contains supplementary material, which is available to authorized users.

## Background

Sugarcane, the highest tonnage crop among cultivated plants, plays a substantial role in the global economy. Nowadays, this crop has gained great importance not only for its traditional use as food (80 % of world’s sugar is produced from sugarcane) but also for ethanol and biomass production. The production of alternative energy sources as well as the establishment of the biorefinery concept has also increased sugarcane world demand rapidly [1]. In order to supply this continuous increasing requirement, the development of new varieties with high biomass and sugar yield is essential.

The modern sugarcane cultivars are interspecific hybrids derived essentially from early crosses between *Saccharum officinarum* (2n = 80, x = 10), a species with high sugar content stalks, and *Saccharum spontaneum* (2n = 40–128, x = 8), a wild and vigorous species resistant to several sugarcane diseases. The initial interspecific hybrids were repeatedly backcrossed to *S. officinarum* clones or to other hybrids in order to recover high sugar content, a process known as “nobilization”. These modern cultivars are highly polyploid and often aneuploid, with chromosome numbers ranging from 100 to 130 [2]. Due to this genetic complexity, the application of both conventional and molecular breeding is a challenge in sugarcane.

Most of sugarcane production regions have their own breeding programs to develop and improve local varieties adapted to their specific environments and agricultural practices. Developing a new sugarcane variety takes on average 12 years [3]. Molecular markers associated with relevant agronomic traits could significantly reduce the time and cost involved in developing new varieties because they could aid in selecting the best parents as well as accelerating the rate of genetic gain in the breeding program. In that sense, association mapping has become widely used to identify molecular markers associated with relevant traits in several crops [4–9]. This method is based on the linkage disequilibrium (LD) between molecular markers and quantitative trait loci (QTL) [10]. The resolution and applicability of association mapping depends on the extent of LD within the population under consideration. The breeding history of sugarcane, consisting of a strong foundation bottleneck followed by a small number of cycles of intercrossing and vegetative propagation, suggest that LD should be extensive, thus a high density of markers may not be needed to detect marker–trait associations [11]. In 1999 [12], and more recently in 2008 [13], the persistence of high LD in modern sugarcane cultivars was confirmed.

The forces generating and/or conserving LD are those that produce allele frequency changes, i.e. population stratification, genetic relatedness, selection, mutation, genetic drift and linkage [10]. With the exception of linkage, all the genetic forces may cause false positive correlation between markers and traits in population-based association mapping approaches. The effects of a structured population in association mapping studies have been well documented and identified as one of the main causes of spurious associations [14–16]. For that reason and considering the often complex relationships among genotypes in breeding populations, it is extremely important to control for population structure in order to effectively decrease type I error rates (i.e. false positives) [17]. For this purpose, a range of statistical methodologies have been developed that include some sort of population or relatedness control using mixed models [16–19].

In addition to controlling for population structure, the availability of both accurate phenotypic data and molecular markers distributed across the genome are critical requirements for the success of association mapping. One of the advantages of this mapping method for plants compared to classical QTL analysis based on balanced mapping populations is that association mapping allows the use of historical phenotypic data sets collected by the breeding programs [5]. Typically, this data come from multiple trials across different environments and years, therefore, statistical analysis such as mixed models are necessary to obtain phenotypic values that best represent the performance of each genotype. Malosetti et al. [19] extended the standard phenotypic analysis of multiple trials by mixed models to arrive at models suitable for association mapping by introducing marker genotype information as random covariates to model the correlation between genotypes.

The recently developed technology of DArT in sugarcane [1] makes it possible to have genome-wide scans of this genetically complex crop, capturing genomic profiles with many thousands of polymorphic markers of several kinds (INDELs, SNPs, methylation changes) [20]. Another molecular marker system recently developed that could also be convenient to detect markers associated with desirable traits is Target Region Amplification Polymorphism (TRAP). These dominant markers enable the identification of polymorphisms in coding regions involved in specific pathways as sucrose metabolism or drought tolerance among others [21, 22].

Information of the marker sequences for DArT is available and could be anchored to the sugarcane genome if sequenced. Several efforts are still ongoing in order to sequence the sugarcane genome which has a high genetic complexity due to its ploidy level. However, considering that i) sugarcane monoploid genome estimated on 930 Mb is similar to the sorghum genome (2n = 2x = 10) estimated on 730 Mb [23]; ii) sugarcane and sorghum both belong to the *Poaceae* family and the same sub-tribu *Saccharinae*, and iii) their high degree of colinearity [24, 25]; the available sequence of sorghum genome becomes an important tool for the analysis of regions of interest in sugarcane.

The goal of this research was to establish an appropriate genome-wide association analysis (GWAS) tool in a sugarcane breeding population, and to find molecular markers associated with high yield of both biomass and sugar stable through successive crop cycles. Therefore, a GWAS mapping within a mixed-model framework following Malosetti et al. [19] was used. Spurious associations were minimized while the power to detect true associations was maximized by considering the possible population structure. A Principal Component Analysis (PCA) from a genotype data set was performed [26] and values obtained from the significant axes for each genotype were used as covariates in the model. In contrast with others sugarcane GWAS studies reported earlier involving yield related traits [27, 28] where analyzes were conducted at plant-cane stage, the novel methodology to analyze multi-QTLs through successive crop cycles used in the present study allowed us to find several markers associated with relevant traits. Results highlighted that this approach could be a valuable tool to assist the improvement of sugarcane and better supply the sugar and biomass demand that has been projected for the upcoming decades.

## Methods

### Plant material and phenotyping

The experimental population consisted on sugarcane clones from the selection panel (Infield Variety Trials, IVT) of the sugarcane breeding program of “Estación Experimental Agroindustrial Obispo Colombres” (SCBP-EEAOC) (i.e. 88 clones, Table 1). IVT are the fourth step of selection of SCBP-EEAOC, where in 2008 a total of 100 clones were planted and thoroughly evaluated in 2009 in order to select potentially new varieties at the following steps. This breeding population consists in genotypes obtained from crosses between the best parents, i.e. with highly productive offspring. To avoid the over-representation of any family, out of the 100 clones, 14 full-sibs were removed to assemble the panel suitable for association mapping. Only some full-sib clones were conserved for not reducing the number of genotypes of the population. The first and second more planted varieties in Tucumán (Argentina) LCP 85-384 and TUCCP 77-42, respectively [29], were also included in the association panel. The IVT were conducted at two locations in Tucumán, Argentina (Additional file 1) during three successive crop cycles. Within each trial, a randomized complete-block design with three replications was used. The individual plot size was 3 rows x 10 m, with an inter-row spacing of 1.6 m. Cane yield (CY) (kg plot^-1^) was evaluated directly by weighing stalks from the full plot in the field during the harvesting season 2009 (plant cane), 2010 (first ratoon), and 2011 (second ratoon). Even though CY was measured in kg plot^-1^ in the present GWAS study, final effects were converted to t ha^-1^ for a better interpretation. In May of each year, sugar content (SC) was estimated from ten randomly chosen stalks from each plot by determining Brixº (percentage of soluble solids, mostly sugars, minerals, and organic acids) and Pol (level of sucrose in stalk juice determined by polarimetry) [30, 31]. SC was determined at the millroom of an EEAOC’s laboratory by using Brixº and Pol, according to the following equation:

**Table 1:** Sugarcane accessions and their parents used in the genome-wide association study of cane yield and sugar content

	Accession	Female parent	Male parent		Accession	Female parent	Male parent
1.	TUC 01-39	LCP 85-384	LCP 82-90	45.	TUC 03-17	Unknown	Unknown
2.	TUC 01-40	TUC 89-5	HOCP 91-552	46.	TUC 03-18	Unknown	Unknown
3.	TUC 01-41	HOCP 85-845	S89-P28	47.	TUC 03-19	Unknown	Unknown
4.	TUC 01-42	TUC 84-31	HOCP 91-552	48.	TUC 03-20	LCP 85-376	HOCP 91-552
5.	TUC 01-43	CP 79-318	HOCP 91-552	49.	TUC 03-21	HOCP 92-648	TUC 77-42
6.	TUC 01-44	CP 79-318	HOCP 91-552	50.	TUC 03-22	HOCP 92-648	TUC 77-42
7.	TUC 01-45	TUC 90-5	HOCP 94-856	51.	TUC 03-23	HOCP 91-555	TUC 91-11
8.	TUC 01-46	HOCP 91-555	TUC 89-30	52.	TUC 03-24	LCP 81-281	TUC 77-42
9.	TUC 01-47	HOCP 92-624	HOCP 91-552	53.	TUC 03-25	L 95-466	TUC 72-16
10.	TUC 01-48	HOCP 93-746	TUC 77-16	54.	TUC 03-26	TUC 89-28	TUC 91-2
11.	TUC 02-27	HOCP 92-631	TUC 93-16	55.	TUC 03-27	HOCP 91-559	HOCP 91-552
12.	TUC 02-29	HOCP 92-675	LCP 82-89	56.	TUC 03-28	CP 65-350	HOCP 93-754
13.	TUC 02-30	TUC 89-32	CP 57-617	57.	TUC 03-29	L 94-433	CP 88-2377
14.	TUC 02-31	FAM 89-604	LCP 85-384	58.	TUC 01-49	Unknown	Unknown
15.	TUC 02-32	RA 89-60	LCP 85-384	59.	TUC 02-63	Unknown	Unknown
16.	TUC 02-34	TUC 87-2	TUC 77-42	60.	TUC 02-64	CP 88-1162	LCP 85-384
17.	TUC 02-35	HOCP 91-555	HOCP 92-64	61.	TUC 02-65	CP 88-1162	LCP 85-384
18.	TUC 02-36	HOCP 93-746	TUC 87-5	62.	TUC 02-67	HOCP 94-806	TUC 89-30
19.	TUC 02-37	TUC 87-2	L 91-264	63.	TUC 02-68	HOCP 94-806	TUC 89-30
20.	TUC 02-38	TUC 87-2	L 91-264	64.	TUC 02-69	HOCP 94-806	LCP 85-384
21.	TUC 02-39	HOCP 91-555	TUC 93-1	65.	TUC 02-70	LCP 85-384	HOCP 83-750
22.	TUC 02-40	HOCP 94-806	TUC 89-30	66.	TUC 03-30	L 89-113	LCP 85-384
23.	TUC 02-41	LCP 85-384	HOCP 83-750	67.	TUC 03-31	TUC 92-3	HO 94-856
24.	TUC 02-42	TUC 91-1	LCP 85-384	68.	TUC 03-32	TUC 92-3	HO 94-856
25.	TUC 02-43	LCP 82-89	HOCP 94-806	69.	TUC 03-33	TUC 92-3	HO 94-856
26.	TUC 02-44	L 90-178	TUC 93-1	70.	TUC 04-1	Unknown	Unknown
27.	TUC 02-45	HOCP 85-845	HOCP 95-961	71.	TUC 04-2	Unknown	Unknown
28.	TUC 02-46	HOCP 85-845	HOCP 95-961	72.	TUC 04-3	TUC 77-42	LCP 85-384
29.	TUC 02-47	HOCP 85-845	HOCP 95-961	73.	TUC 04-4	TUC 93-87	TUC 77-42
30.	TUC 02-48	HOCP 85-845	HOCP 95-961	74.	TUC 04-5	TUC 93-8	LCP 85-384
31.	TUC 02-49	HOCP 85-845	HOCP 95-961	75.	TUC 04-6	TUC 93-98	TUC 87-3
32.	TUC 02-50	Unknown	Unknown	76.	TUC 04-7	LCP 85-384	TUC 77-42
33.	TUC 02-51	Unknown	Unknown	77.	HOCP 00-950	HOCP 93-750	HOCP 92-676
34.	TUC 02-52	Unknown	Unknown	78.	TUC 01-55	HOCP 92-624	TUC 72-716
35.	TUC 02-53	Unknown	Unknown	79.	TUC 01-56	HOCP 85-845	HOCP 92-631
36.	TUC 02-54	Unknown	Unknown	80.	TUC 02-71	TUC 89-29	HOCP 92-631
37.	TUC 02-55	Unknown	Unknown	81.	TUC 03-34	L 89-113	TUC 87-3
38.	TUC 02-56	Unknown	Unknown	82.	TUC 03-35	HOCP 92-631	TUC 72-16
39.	TUC 02-57	Unknown	Unknown	83.	TUC 03-36	TUC 93-98	RA 89-604
40.	TUC 02-58	RA 87-2	L 91-264	84.	TUC 03-37	TUC 92-3	HO 94-856
41.	TUC 02-59	TUC 89-32	LCP 82-89	85.	TUC 03-39	HOCP 92-648	TUC 87-5
42.	TUC 02-60	LCP 94-806	LCP 85-384	86.	TUC 03-43	HOCP 92-675	TUC 71-7
43.	TUC 02-61	HOCP 91-555	TUC 95-23	87.	LCP 85-384	CP 77-310	CP 77-407
44.	TUC 02-62	TUC 89-28	L 94-424	88.	TUCCP 77-42	CP 71-321	US 72-19

$$ \mathsf{S}\mathsf{C}\% = \mathsf{0}.\mathsf{98} \times \mathsf{p}\mathsf{o}\mathsf{l}\ \%\ \hbox{-}\ \mathsf{0}.\mathsf{28} \times \mathsf{brix}\ \% $$

[32]

### Statistical analysis for the phenotypic data

Field trials were analyzed for each harvesting season independently using the following mixed model:

$$ {\mathsf{y}}_{\mathit{\mathsf{i}}\mathit{\mathsf{j}}\mathit{\mathsf{k}}}=\mathsf{\mu}+{\mathsf{G}}_{\mathit{\mathsf{i}}}+{\mathsf{S}}_{\mathit{\mathsf{j}}}+{{\mathsf{B}}_{\mathit{\mathsf{k}}}}_{\left(\mathit{\mathsf{j}}\right)}+\mathsf{G}{\mathsf{S}}_{\left(\mathit{\mathsf{i}}\mathit{\mathsf{j}}\right)}+{\mathsf{\varepsilon}}_{\mathit{\mathsf{i}}\mathit{\mathsf{j}}\mathit{\mathsf{k}}} $$

where y_*ijk*_ is yield of genotype *i* at location *j* and block *k*; μ is the overall mean; G_*i*_ is the *i*-th genotype fixed effect with *i =* 1,…,*g*; S_*j*_ is the *j*-th location random effect with *j* = 1,…,*s* and S_j_ ~ N(0, σ^2^_S_); B_*k*(*j*)_ is the *k*-th block random effect at location *j* with *k* = 1,…,*n* and B_k(j)_ ~ N(0, σ^2^_B_); GS_(*ij*)_ is the genotype *i* by location *j* interaction random effect with GS_(ij)_ ~ N(0, σ^2^_GS_); and ε_*ijk*_ is the random error associated with observation y_*ijk*_. Comparison through harvesting seasons is particularly interesting since dynamics and characteristics of plant-cane bud sprouting and growth are different from those of ratoon crop [33]. Therefore, different genome regions would be implied in yield of both cane and sugar, through different crop ages. The estimated means (Best Linear Unbiased Estimator, BLUE) obtained from this model for CY and SC of all genotypes were used for the association mapping analysis. The analysis was performed using PROC MIXED in SAS software 9.0 (SAS Institute 2004). A mixed model for association mapping was used later (described below) and therefore, BLUEs instead of BLUPs were used as genetic values for the accessions to avoid double-shrinking [34–38]. Pearson correlation of genotypic means was estimated between traits in R software [39]. Broad-sense heritability (H^2^) at an experimental level was calculated on a genotype mean basis for each trait and at each location as the ratio of genotypic to phenotypic variance, using the components of variance obtained from a model adjusted as follows:

$$ {H}^2=\frac{\sigma_G^2}{\sigma_G^2+{\sigma}_{\varepsilon}^2/r} $$

where *σ*_*G*_^2^ is the genetic variance, *σ*_*ε*_^2^ the residual variance and *r* the number of replicates [40].

### Genotyping

DNA was extracted from frozen leaf tissue following the Diversity Arrays Technology (DArT) Pty Ltd (Yarralumla, Australia) protocol [41]. The quality and quantity of DNA were verified on a 0.8 % agarose gel. All clones were genotyped using DArT [1] and TRAP markers [21, 22]. DArT genotyping of the population mapping was carried out by DArT Pty Ltd with the Sugarcane High Density 1.0 array. This service involves two methods of complexity reduction (both based on *PstI*-based methyl filtration) against the array containing 7680 probes. TRAP genotyping was carried out according to [22] with minor modifications. All PCR reactions were carried out in our lab and performed in a Bio-Rad My clycler Termalcycler (Hercules, CA, USA) in 5 μl reaction containing 50 ng DNA sample, 10X reaction buffer (Fermentas, Spain, EU), 2.5 mM MgCl_2_ (Fermentas), 0.088 mM of each dATP, dTTP and dGTP, 0.072 mM of dCTP, 0.16 μM of each primer (Table 2), and 0.5 U of Taq DNA polymerase (Fermentas). Different concentrations of Cy5.5-dCTP (GE Healthcare, Buckinghamshire, UK) were included in the reaction depending on the primer combination (Table 2). Amplifications were performed by initially denaturing the template DNA at 94 °C for 2 min, followed by five cycles at 94 °C for 45 s, 35 °C for 45 s, and 72 °C for 1 min, 35 cycles at 94 °C for 45 s, 50 °C for 45 s, and 72 °C for1 min, and a final extension step at 72 °C for 7 min. Loading dye was added and 0.3 μl PCR products were separated on a 25 cm polyacrylamide gel (Amersham Biosciences) (0.25 mm thick) in a LI-COR 4300 DNA Analyzer (LICOR Biosciences, Lincoln, NE, USA) according to manufacturer’s instructions. Images were captured with slow scan laser at 700 nm and analyzed with the SAGA^TM^ software (LICOR Biosciences). The product sizes were determined by comparison with molecular weight marker LI-COR IRDye 50–700 bp Size Standard (LICOR Biosciences). TRAP markers, classified as 1 (presence) or 0 (absence), and the binary data from DArT were used for association analysis. All markers with a minor allele frequency (MAF) lower than 0.1 were excluded from the GWAS analysis.

**Table 2:** Conditions for sugarcane TRAP genotyping used in the GWA study of sugarcane breeding population

TRAP	Primer forward		Primer reverse		^a^Cy5.5-dCTP [μM]
	Name	Sequence (3′-- > 5′)	Name	Sequence (3′-- > 5′)	
T14	SuPS/ Sucrose phosphate synthase	CGACAACTGGATCAACAG	Arbi-2	GACTGCGTACGAATTGAC	0.8
T15	SuPS/ Sucrose phosphate synthase	CGACAACTGGATCAACAG	Arbi-3	GACTGCGTACGAATTTGA	0.5
T17	DirH/ Dirigent protein	TGGAGATTTTTGGAGGAAC	Arbi-2	GACTGCGTACGAATTGAC	0.5

^a^Final concetration of Cy5.5-dCTP in reaction

### Genetic diversity and population structure

All polymorphic DArT and TRAP markers scored on the 88 sugarcane accessions were used to estimate genetic relationship among clones. Genetic dissimilarities between all pairwise combinations of clones were calculated using the Dice index [42]. Then, a Neighbor Joining tree was built from the matrix of pairwise dissimilarities using the Darwin software V.5.0.158 [43].

In order to detect and correct for population structure, a PCA was carried out using a subset of 107 DArT markers. All the available markers were not included in this analysis mainly because using the same markers to estimate population structure and then including them in the model to test for an association could create a dependency among terms in the model absorbing some of the QTL effects [44]. The markers used for PCA were sampled according to their position on different Linkage Groups of the Homology Groups of a sugarcane map recently published [45].

### GWAS analysis

A mainstream mixed model GWAS analysis was conducted following [19] and [46]. Associations between molecular markers and quantitative traits were determined following the general linear mixed model for each year:

$$ \mathsf{Y}=\mathsf{X}\mathsf{\beta }+\underline {\mathsf{Q}\mathsf{\upsilon }}+\mathsf{e} $$

where Y is the phenotypic means vector (i.e. BLUEs from field analysis), X is the incidence matrix of molecular markers, β is the vector of parameters related to the simple regression of the markers on the phenotypes, Q are the eigenvectors of the significant axes of the PCA matrix, υ is a vector of predicted values of population structure, and e is the vector of random errors. The PCA scores were used in the model as random components following [19] and [46]. Modeling population structure as random effects not only does the relatedness matrix capture population structure, but also encodes a wider range of structures, including cryptic relatedness and family structure [36, 47, 48]. The significant PC axes included in the model were determined with the Tracy-Widom statistic [46]. The analyses were performed using R-code developed by the author’s with modifications from the *emma* [49] and *GAPIT* [50] packages and recently published [40] using the R software 3.0.0. The code will be uploaded to the R-Cran repository as *mmQTL* package [51]. Briefly, a two-step approach was followed to arrive to a multi-QTL model. First, a marker-by-marker scan of the genome was conducted to identify significant marker-trait associations with a false-discovery rate (FDR) (α = 0.05) to control for multiple testing. Since a large number of significant marker-trait associations were found, and to report the more relevant QTL, a second pruning of markers with a more stringent FDR P-value (0.01) was conducted. Second, all significant markers were fitted in a single final multi-QTL model adding markers at a time in a stepwise-forward selection manner to control for residual QTL and to identify QTL following [52–54]. The Wald statistic with a liberal P-value < 0.01 following [19, 36] was used for this model.

QQ-plots assuming a uniform distribution of P-values under the null-hypothesis of no-QTL (i.e., Schwederand Spjøtvoll plots; [55]) were used to evaluate the models. Briefly, the observed P-values values are plotted against the expected theoretical values (i.e. cumulative density function) for a uniform distribution. This is standard methodology to evaluate the models ability to control for spurious association [17, 36, 56]. These analyses were also performed in R statistical software.

### Analysis of sugarcane DArT marker sequences associated to important traits

Sequences from sugarcane DArT markers significantly associated with CY or SC at least in 2 years of study and DArT markers significantly associated with a trait in the multi-QTL model that resulted in highest Allelic Substitution Effect (ASE) were used to determine their similarity and position on the sorghum genome. This was conducted by using BLASTN 2.2.22 [57] on non-redundant databases of sorghum sequences with different algorithms. First, “Megablast” was employed to identify query sequences. In the cases where no significant similarity was found, a second algorithm “Discontiguous megablast” was chosen since it uses an initial seed that ignores some bases and is intended for cross-species comparisons. Finally, when no significant similarity was found using the second algorithm, BLAST was performed using “blastN”.

## Results

### Phenotypic data, molecular markers, panel diversity and population structure

The 88 sugarcane clones used in this study were phenotyped by SCBP-EEAOC for CY and SC during 2009, 2010 and 2011 and genetically characterized by DArT and TRAP markers. The BLUE values obtained with the adjusted model, described above, were 48 to 85 t ha^-1^ for CY and 9.2 to 10.9 % for SC (Table 3 and Additional file 2). The genetic correlations observed between years for CY were 0.60 for 2009 and 2010, 0.78 for 2010 and 2011, and 0.50 between 2009 and 2011. Meanwhile, genetic correlations observed between years for SC were 0.40 for 2009 and 2010, 0.72 for 2010 and 2011, and 0.46 between 2009 and 2011. There were low correlations between CY and SC across years (-0.06, -0.24 and -0.14 for 2009, 2010 and 2011, respectively), being only significant (*P*-value <0.05) correlation among CY 2010 and SC 2010 (Additional file 3). Results of broad-sense heritability for both trait and location are presented in Table 4. CY was under strong genetic control, since estimates of broad-sense heritability were high, ranging from 0.51 to 0.84. Estimates of H^2^ for SC were also high (from 0.55 to 0.80), with the only exception for SC 2010 with a moderate value of H^2^ of 0.30. This high estimates of heritability indicated that the field trials produced good-quality data for the association study.

**Table 3:** Descriptive statistics of cane yield (CY) and sugar content (SC) from field trial of all genotypes evaluated in the GWA study

	CY (t ha^-1^)		SC (%)	
	Mean	CV	Mean	CV
Plant-cane (2009)	47.70	0.20	9.22	0.08
First ratoon (2010)	75.14	0.13	10.62	0.06
Second ratoon (2011)	84.95	0.12	10.88	0.06

*CV* coefficient of variation

**Table 4:** Broad-sense heritability (H^2^) at each location and at each crop cycle for Cane Yield and Sugar Content

Crop cycle	Traits	Location	
		Cerco Represa	Santa Ana
Plant	Cane Yield 2009	0.747	0.513
	Sugar Content 2009	0.666	0.618
Ratoon 1	Cane Yield 2010	0.758	0.649
	Sugar Content 2010	0.553	0.301
Ratoon 2	Cane Yield 2011	0.699	0.835
	Sugar Content 2011	0.800	0.596

Out of the 7680 probes evaluated in the DArT array, 1642 markers were informative (i.e. polymorphic, with a MAF higher than 0.10). Out of the 177 TRAP markers evaluated, only 103 markers were included in the GWAS and 74 were excluded because the MAF was lower than 0.1. Among the 1642 informative DArT markers, 258 were mapped on the recently published sugarcane genetic map [45].

**Fig. 1:**
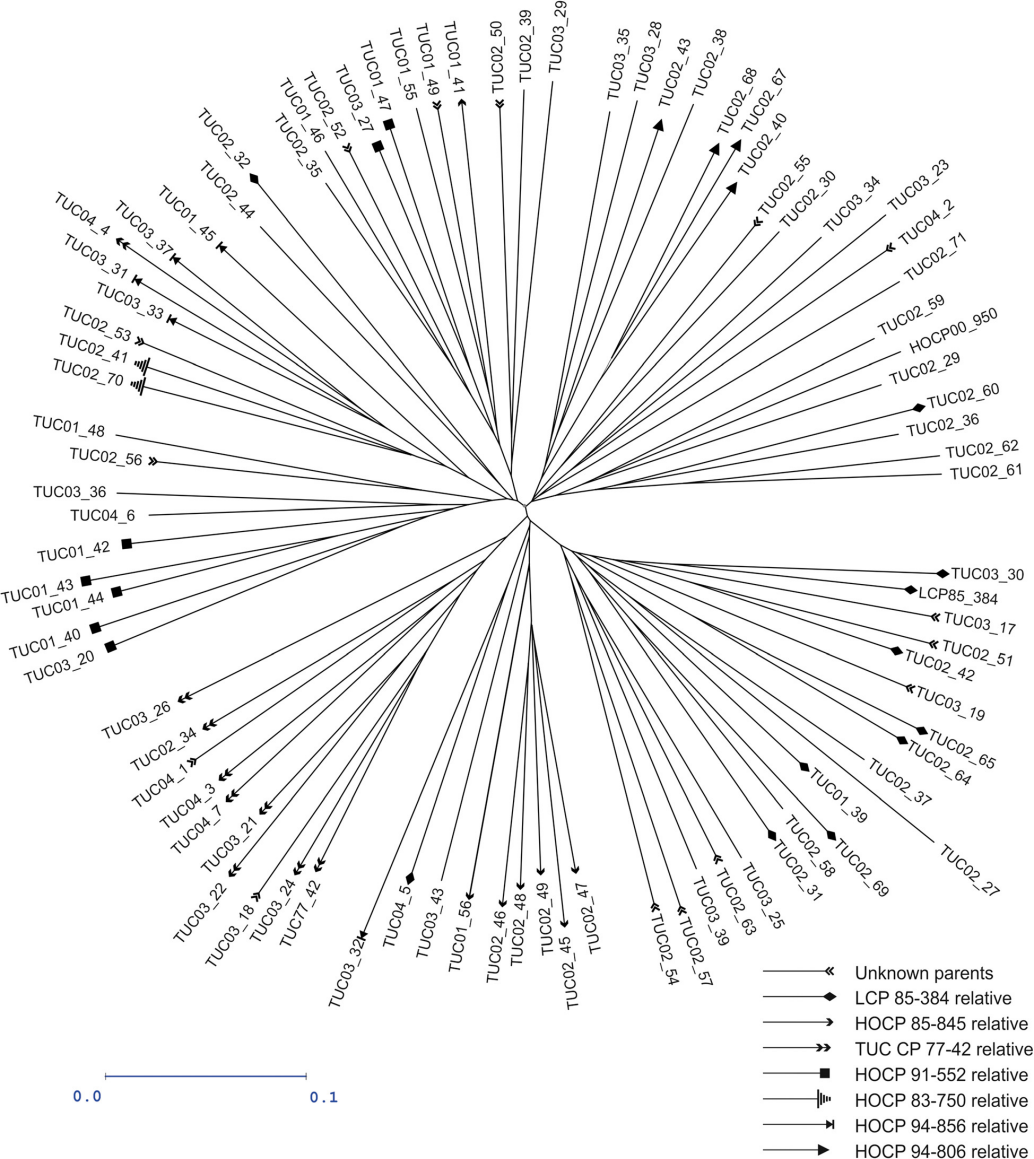
Neighbour-joining tree based on the Dice dissimilarity index calculated from 1745 polymorphic markers data (103 TRAP and 1642 DArT) assembling the 88 sugarcane genotypes

**Fig. 2:**
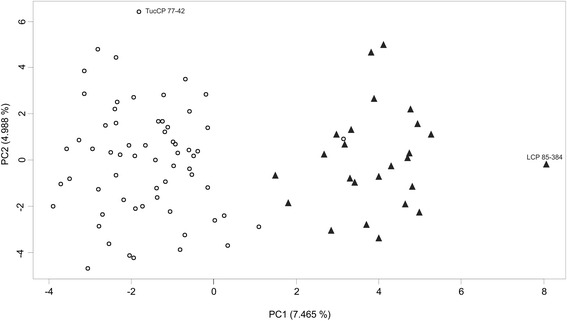
The top two axes of variation of 88 sugarcane clones studied resulting of Principal Component Analysis by using 107 DArT markers distributed across the genome. The percentage of variation represented by each component is in parentheses. Accessions are colored according to their parentage with LCP 85-384. Progeny of LCP 85-384 are in black triangle (▲); the remaining genotypes are in empty circles (◯)

Diversity analysis using all the informative TRAP and DArT markers revealed no particular structure in the mapping population (Fig. 1 and Additional file 4; http://dx.doi.org/10.5061/dryad.mv88m). The most closely related clones (parent–descendant or full-sib) were grouped in the same area of the neighbor-joining tree. However, they do not form outstanding branches. Surprisingly, there were two exceptions where full-sib clones were located in different branches, i.e. TUC 02-38 and TUC 02-37 whose genealogical records indicate that they are descendant from the same parents; and TUC 03-32 that would be full-sib with TUC 03-31, TUC 03-33, TUC 03-37 and TUC 04-4, and grouped separately from the rest. At the most distant branch, located at the lower right portion of the tree, grouped LCP 85-384 and most of the clones derived from this variety. At the lower center position of the tree, clones derived from HOCP 85-845 were grouped. Then, at the lower left portion of the tree, TUCCP 77-42 and clones derived from this variety were located. On the other hand, the first three axes of the PCA using 107 DArT markers distributed across the sugarcane genome were significant following the Tracy-Widom statistic. The PCA scores for each genotype at each axes were included as random covariates in the GWAS model to model the variance-covariance matrix among genotypes. The first two axes explained 7.47 and 4.99 % of the total variation, respectively (Fig. 2). The first axis could be associated to filial relations; where two groups seems associated to LCP 85-384 offspring (right side of the PC1 axis) and non-LCP 85-384 offspring (left side of the PC1 axis). At PC2 level, TUCCP 77-42 variety was distant from the rest of the genotypes. Results showed at Fig. 2 are congruent with those previously mentioned in Fig. 1, since clone descendant from LCP 85-384 were detached from the rest of genotypes.

### GWAS analysis

GWAS analysis was conducted by using 1638 discrete markers (1535 DArT and 103 TRAP). QQ-plots of *P*-values showed that population structure was properly accounted for by using a stratified selection of markers to correct for population structure as random effect (Additional file 5). In the present study, 43, 42 and 41 markers significantly associated (FDR α = 0.01) with CY in 2009 (cane plant), 2010 (first ratoon) and 2011 (second ratoon), respectively, were found. In addition, 38, 34 and 47 significant marker-trait associations for SC were detected, in 2009 (cane plant), 2010 (first ratoon) and 2011 (second ratoon), respectively (Additional file 6). Certain stability across crop-cycles was observed since twenty markers were found to be associated with CY in 2 years of study, being the coincidence between 2010 and 2011 (first and second ratoon) more frequent. For SC, one marker-trait association was found significant for the 3 years of study, while twelve markers presented association for 2 years. These association were also more frequent when 2010 and 2011 years were involved (Table 5). Mostly markers associated with one trait were not associated with the other; however, four markers were associated with both traits (M54 for CY-2010, CY-2011 and SC-2011; M58 for CY-2010, CY-2011 and SC-2011; M173 for CY-2010, SC-2010 and SC-2011; and, M188 for CY-2010, SC-2010 and SC-2011).

**Table 5:** Summary of results found for markers associated with traits of interest at least in two years of study and comparison with sorghum genome

*Locus*	Cane Yield			Sugar Content			DArT sequence size (pb)	BLAST algorithm ^d^	Result from alignament with *S. bicolor*	Identity	Expect	Locus tag	Sorghum chromosome	GenBank ID
	2009	2010	2011	2009	2010	2011								
M151	*		*				748	blastn	hypothetical protein	49/64(77 %)	0.001	Sb01g041060	1	XM_002468130.1
M97		*	*				**456**	**m blast**	**hypothetical protein**	**419/459(91 %)**	**9.00E-173**	**Sb02g004780**	**2**	**XM_002461519.1**
M41					*	**	263	blastn	hypothetical protein	45/58(78 %)	0.25	Sb02g028450	2	XM_002460412.1
M91	*	*					404	dm blast	hypothetical protein	68/68(100 %)	1.00E-27	Sb03g004640	3	XM_002457229.1
M64				*	*	*	**291**	**m blast**	**hypothetical protein**	**287/291(99 %)**	**6.00E-143**	**Sb03g035700**	**3**	**XM_002456357.1**
M78				*		*	474	m blast	hypothetical protein	79/83(95 %)	4E-30	Sb04g025280	4	XM_002452369.1
M125		*	*				1048	dm blast	hypothetical protein	87/121(72 %)	9E-08	Sb05g000890	5	XM_002448838.1
M95		*	*				545^a^	dm blast	hypothetical protein	122/146(84 %)	2E-37	Sb06g014780	6	XM_002447689.1
M50		*	*				545^a^	dm blast	hypothetical protein	122/146(84 %)	2E-37	Sb06g014780	6	XM_002447689.1
M120		*	*				**457**	**m blast**	**hypothetical protein**	**407/435(94 %)**	**0**	**Sb06g000450**	**6**	**XM_002445961.1**
M57					*	***	60	blastn	hypothetical protein	20/21(95 %)	0.34	Sb06g000690	6	XM_002445988.1
M189		*	*				355	blastn	hypothetical protein	18/18(100 %)	1.2	Sb06g025190	6	XM_002448276.1
M46	*	*					371^b^	m blast	hypothetical protein	119/121(98 %)	4E-54	Sb07g020840	7	XM_002444326.1
M30	*	**					384^b^	m blast	hypothetical protein	119/121(98 %)	4E-54	Sb07g020840	7	XM_002444326.1
M71		*	*				425	blastn	hypothetical protein	59/73(81 %)	3.00E-09	Sb08g022800	8	XM_002443601.1
M58		**	*			*	**566** ^**c**^	**m blast**	**hypothetical protein**	**354/371(95 %)**	**7E-168**	**Sb09g002270**	**9**	**XM_002439164.1**
M54		**	*			*	**566** ^**c**^	**m blast**	**hypothetical protein**	**354/371(95 %)**	**7E-168**	**Sb09g002270**	**9**	**XM_002439164.1**
M168				*	*		**384**	**m blast**	**hypothetical protein**	**368/384(96 %)**	**7E-176**	**Sb09g015250**	**9**	**XM_002440847.1**
M59		*	*				468	blastn	hypothetical protein	23/25(92 %)	0.13	Sb09g007900	9	XM_002439437.1
M193		*	**				604	blastn	hypothetical protein	18/18(100 %)	2.1	Sb09g014225	9	XM_002439535.1
M153					*	*	753	dm blast	hypothetical protein	85/87(98 %)	6E-35	Sb10g006890	10	XM_002437984.1
M14					*	*	427	blastn	hypothetical protein	23/24(96 %)	0.43	Sb10g010770	10	XM_002438218.1
M188		*			*	*	578	blastn	hypothetical protein	29/34(85 %)	0.59	Sb10g023910	10	XM_002437224.1
M45				**		*	749	blastn	alpha kafirin	79/98(81 %)	3.00E-18	unassigned		Y17556.1
M181				**		*	749	blastn	alpha kafirin	46/46(100 %)	5E-16	unassigned		Y17556.1
M108	*		*				488	m blast	alpha kafirin	61/62(98 %)	7E-23	unassigned		Y17556.1
M173		*			*	*	662	blastn	alpha kafirin	39/41(95 %)	5E-09	unassigned		Y17556.1
M5					*	*	na							
M86					*	*	na							
M32		*	*				na							
M198	*		*				na							
M197	**	*					na							
M203	*	*					na							

*na* not available sequence

*FDR P*-values: * *p* < 0.01; ** *p* < 0.001; and *** *p* < 0.0001

^a, b, c^indicate same nucleotide sequence for two diferent DArT marker

^d^megablast (m blast), discontiduous mega blast (dm blast) or blastn

Data in bold = more significant alignment i.e. larger sequence size with high identity and lower Expected value

A multi-QTL model by year was constructed with markers significantly associated with each trait. Considering the 3 years, 23 markers were significant in the multi-QTL for CY while 21 remained significant in the multi-QTL for SC (Table 6). For CY, markers M100, M120, M140, M200 and M202 had allelic substitution effect (ASE) larger than 8.33 t ha^-1^. For SC, M28, M51 and M171 had ASE larger than 0.70 %. Marker M64 was detected in more than 1 year in the multi-QTL model (SC 2010 and 2011). The effect of this marker was the same in the 2 years of association and 57 % of the genotypes analyzed had the favorable allele for this marker.

**Table 6:** Significant markers associated to cane yield and sugar content and their allelic substitution effect (ASE) in the multi-QTL model for the sugarcane GWAS panel

Cane Yield								
2009			2010			2011		
*locus*	ASE^a^	*P*- value^b^	*locus*	ASE^a^	*P*- value^b^	*locus*	ASE^a^	*P*- value^b^
M155	4.11	0.00859	M120	-8.70	0.00001	M100	8.69	0.00013
M17	5.35	0.00114	M140	9.91	0.00002	M105	-4.78	0.00692
M185	7.18	0.00012	M188	4.19	0.01031	M131	6.36	0.00028
M200	-8.36	0.00001	M189	5.52	0.01048	M145	-4.19	0.02756
M30	-4.41	0.01525	M197	7.29	0.00007	M166	4.17	0.01292
M35	-6.00	0.00464	M59	-4.71	0.00441	M193	-4.71	0.01644
			M72	-8.22	0.00002	M202	8.92	0.00001
						M47	5.51	0.00361
						M98	-7.47	0.00694
						M99	6.65	0.01779
Sugar Content								
2009			2010			2011		
*locus*	ASE^a^	*P*- value^b^	*locus*	ASE^a^	*P*- value^b^	*locus*	ASE^a^	*P*- value^b^
M147	0.3	0.04552	M103	-0.3	0.00728	M101	-0.53	0.00001
M156	-0.54	0.00011	M124	-0.31	0.00873	M15	0.42	0.00293
M181	0.55	0.00011	M153	0.42	0.01627	M150	-0.22	0.04433
M28	-0.81	0.00005	M171	-0.76	0.0000002	M194	0.29	0.01277
			M177	-0.38	0.00374	M205	-0.38	0.00293
			M183	-0.23	0.06801	M51	0.71	0.00006
			M206	0.33	0.01723	M64	-0.48	0.00017
			M5	0.4	0.00181	M86	0.25	0.02331
			M64	-0.48	0.00003			

^a^Allele Substitution Effect. Values for cane yield were transformed from Kg plots^-1^ to t ha^-1^ after of the analysis for a better depiction. Values for Sugar Content are expressed in %. Negative sign indicate that the absence of the marker is the desirable allele

^b^ *P*-value of individual markers in the multi-QTL model

### Sugarcane DArT markers sequences on sorghum genome

The 27 available sequences of DArT markers significantly associated with a trait in at least 2 years of study were blasted to the sorghum genome sequence database (Table 5). When the sequences of sugarcane DArT markers were analyzed, three of them were found to present the same nucleotide sequence. This was useful as internal control because genotypes presented the same configuration (absence or presence) for markers with the same sequence. Most of alignments involved sequences of hypothetical proteins of sorghum showing a high identity and low e-values. Noticeably M120 showed a high identity (94 %) with a sorghum sequence located on chromosome 6 with an e-value of 0, indicating that there is no probability of alignments with scores equivalent to or better in a database search by chance.

Similarly, sequences of DArT markers significantly associated with a trait in the multi-QTL model that resulted in highest ASE value (M100, M120 and M140 for CY; M28, M51, M64 and M171 for SC) were blasted to the sorghum genome sequence database. Results are shown in Table 7, markers M120 and M64 were not included since they were already shown in Table 5. Other markers with highest ASE value that were not blasted since no sequence information is available, were TRAP markers M200 and M202, which derive from T15 and T17 amplifications respectively (see Table 2). Interestingly, some DArT markers, mainly associated with SC, showed high identity with an alpha kafirin protein that it is involved in the storage of nutritious substrates.

**Table 7:** Results of alignments of markers associated with cane yield and sugar content with higher value of allelic substitution effect (ASE) in the multi-QTL model for the sugarcane GWAS panel against *Sorghum bicolor* sequences

Trait	Marker	DArT sequence size (pb)	BLAST algorithm^a^	Result from alignment with *S. bicolor*	Identity	Expect	Sorghum chromosome	Accesion N°
CY 2010	M140	487	blastn	Sorghum bicolor clone BAC 88 M4, complete sequence	100/132 (76 %)	7.00E-18	8	AY661656.1
CY 2011	M100	318	blastn	Sorghum bicolor putative cytochrome P450-like protein	73/95 (77 %)	2.00E-10	1	AF466201.1
SC 2009	M28	470	dm blast	alpha kafirin	59/62 (95 %)	1.00E-19	unassigned	Y17556.1
SC 2010	M171	711	m blast	alpha kafirin	74/86 (86 %)	5.00E-21	unassigned	Y17556.1
SC 2011	M51	579	dm blast	Sorghum bicolor hypothetical protein, mRNA	74/94 (79 %)	2.00E-14	6	XM_002448117.1

^a^ megablast (m blast), discontiduous mega blast (dm blast) or blastn

## Discussion

In the last decade, several approaches tested in plant genetics have allowed the precise identification of “desirables” alleles at molecular level. In the most recent years, the development of association mapping for this purpose has gained large importance. In this work, association mapping was used in sugarcane to identify molecular markers associated with both sugar and biomass yields. The quantitative nature of both traits and the polyploid genome of this crop make the use of association mapping a great challenge compared to other studies conducted for crops with less complex genomes. Even considering that, in the present study we were able to detect QTL for both traits, which are consistent across harvesting seasons.

### Population mapping

Several studies suggest that the use of elite germplasm could be useful for association mapping [58–60], although there are only a few approaches conducted with this type of population in plant crops (see [60] for a review). In order to take advantage of the available large phenotypic data accumulated from replicated field experiments over locations and years for the SCBP-EEAOC, association mapping was conducted over accessions of its current elite breeding pool (genotypes of the advanced yield trails). All genotypes characterized in the present study were planted and evaluated at the same time obtaining both balanced data across environments and an extensive phenotyping. Therefore, although population sizes are relatively small, the high quality extensive phenotyping provides a reasonable foundation for the GWAS study. Small population sizes would result in decreased power to detect QTL [61, 62] and increased false-positive rate [63, 64]. However, assembling large populations in sugarcane could be a challenge mainly because of the phenotyping requirements and the relative size of the breeding programs. Furthermore, exploring diverse germplasm not adapted to local conditions and with strong population structure could hinder the QTL detection due to the additional challenge of modeling such population structure [65]. Additionally, the mapping approach (i.e., candidate-gene or genome-wide), the relatedness of the individuals, the extent of LD, and the number of markers will determine the optimal population size in GWAS studies [60]. Finally, population sizes close to 100 have been used elsewhere as a first approach to QTL mapping in other species [66–70]. Recent sugarcane GWAS studies included 189 and 183 individuals [27, 71]. However, since the experimental population in the present study was a representative sample of the population to which inference is desired, it is expected that the information obtained from the association study will be useful and readily applicable to local crop improvement [6].

### Controlling population structure

The presence of subpopulations in the mapping population creates a challenge for association studies. Several methods have been proposed for dealing with false positives related to population structure [16–18]. In that sense, many studies conducted especially with small datasets and diploid organisms implemented the method proposed in the freely available software Structure [16]. However, in the case of sugarcane considering its complex polyploid genome, several assumptions are not fulfilled for the use of Structure; therefore, the applicability of this algorithm may be limited in sugarcane [72]. For example, in a previous study in sugarcane [73], when population structure was taken into account by using Structure, arbitrary subpopulations of the genotypes were observed; however, as there were no clear discontinuities in the population, this algorithm failed to conclusively group the population [28]. In the present study a GWAS mapping was applied within a mixed-model framework according to [19] and [46]. Spurious associations were controlled while the power to detect true associations was maximized by using a PCA as a random component to control for population structure [19, 36, 46–48]. When PCA as a random component is included in the analysis, the large population structure is captured with the first few axes that account for most of the variation while the more subtle relationships among individuals are captured by the remaining significant axes.

Population structure was inferred with an independent set of markers to avoid dependency among terms in the model and to prevent the structure from absorbing the QTL effects from the model [44, 46]. A sub-set of available markers to infer population structure has been used in other studies [44], including sugarcane [27]. Gouy et al. [27] used a sub-set of the available markers to ensure genome coverage and avoid over-representation of genomic regions. The sugarcane DArT-based map recently published [45] was used to sample independent markers of each linkage group. Furthermore, QQ-plots of *P*-values showed that population structure was properly accounted for by using a stratified selection of markers to correct for population structure as random effects (Additional file 5). On the other hand, using a random selection of markers without accounting for marker position failed to properly account for population structure (data not shown). Additionally, the grouping observed at PC1 has a biological interpretation, reflecting genetic variation among progeny (Fig. 2). For instance, the right-hand side of the plot includes cv. LCP 85-384 and its progeny; while the left-hand side represents the remaining genotypes. This was also found in other studies, where LCP 85-384 was genetically more distant to modern varieties [74–76]. Cultivar LCP 85-384 is a BC_4_ derived line of *S. spontaneum* US 56-15-8 and therefore have a strong wild genetic component [74]. TUCCP 77-42, another variety with a strong wild genetic component (BC_1_ of *S. spontaneum* SES 147B), was distant from the rest of the genotypes at PC2 level (Fig. 2). These results showed enough evidence of the ability of these few markers (107) used in the PCA to reveal the genetic background of genotypes. Furthermore, most of the structure found in these genotypes seems to come from subtle kinship relationships more than large-scale population structure. Our method properly accounted for these relationships.

### Sugarcane GWAS

Association studies are becoming a popular strategy for unraveling the genetic underlying complex traits. The first association mapping studies conducted in sugarcane have focused on genome-wide approaches attempted at looking for associations between disease resistance and molecular markers [27, 71, 73, 77, 78]. Few reports were found [27, 28] involving associations between molecular markers and traits related with cane and sucrose yield and/or yield components. Wei et al. [28] conducted a study where field-data for cane yield (t ha^-1^) and commercially extractable sucrose content were obtained in plant-cane. However, one of the major concerns in order to find markers contributing to yield during several ages is the repeatability of the marker–trait associations across harvesting seasons (mainly for ratoons). Another study including sucrose yield and yields components, among other traits, was carried out by Gouy et al. [27], obtaining plant-cane phenotypic data from trials planted during different season or years and locations. However, only a few marker-trait associations were detected for the traits analyzed. In that sense, in the present study several markers (20) were found to be associated with CY in at least 2 years, being more frequent the coincidence among first and second ratoon. Sequences of four markers (M58, M54, M97 and M120) showed very high similitude and low e-value with coding sequences of *Sorghum bicolor*. Sequences of M58 and M54 were found to be the same, and they presented high identity and low e-value with a sequence located on chromosome 9 from *Sorghum bicolor*, where QTLs for plant height and tiller number were previously found [79]. Sequence of M97 was located on chromosome 2 of *Sorghum bicolor*, were QTLs for stem diameter and plant height were previously reported [80] and validated later [79]. Regarding M120 marker, whose effect was 8.70 t ha^-1^ in the multi-QTL model, the sequence of this marker presented high similitude with a *Sorghum bicolor* sequence located on chromosome 6, where QTLs for stem biomass yield, plant height and tiller number were reported in different studies (see [79]).

For SC, one marker-trait association was found significant for the 3 years of study. This marker, M64, showed high identity (99 %) and low e-value (6,00 E-143) with a sequence located in chromosome 3 from *Sorghum bicolor*, where several QTL related to sugar content were previously reported (Glucose content [80]; Sugar content [81]; Brixº, Juice Sugars g L^-1^ and Juice Sucrose g L^-1^ [82]) and validated [79]. Moreover, in our multi-QTL model, M64 showed the same marker effect (-0.48 %) in two consecutive years (2010 and 2011), indicating that a negative selection for this marker could increase SC.

The highly conserved sequences found in the sorghum genome confirm the usefulness of this database to study regions of interest in sugarcane genome. Sequence of marker M120 presented 407 identical nucleotides with an e-value of zero, suggesting that this is a region shared by sugarcane and sorghum. Other sugarcane sequence markers were also significantly similar to sorghum, which is in agreement with previous studies [24, 25, 83] that reported a high gene microlinearity between sorghum and sugarcane.

Even though this GWAS study is mostly focused on exploring the entire genome with DArT makers, also TRAP markers that targeted to coding regions were employed. This information resulted useful in finding regions controlling traits of interest since three of the 103 TRAP markers used for association analysis were significantly associated with CY in 2 years of study.

It is important to highlight the challenge in finding strong marker-trait associations in complex polyploid species using dominant markers. It is well known that this type of markers are less informative than co-dominant ones, especially in polyploids, because copies of homologous chromosomes “dilute” the polymorphisms. When the markers are evaluated with a binary system, they are scored as 0 for the absence of the allele, or 1 for the presence of at least one copy of the allele. This constitutes one intrinsic limitation of the method that is associated with overlooking ploidy level [84]. In that sense, further research need to be conducted to investigate the establishment of associations between continuous data obtained from DArT markers and allele dosage, instead of binary data. This would probably increase the number of markers associated with characteristics of interest. Moreover, considering that the study was carried out on a panel of sugarcane varieties and elite lines, most favorable alleles would probably be fixed; however, no single variety has all favorable alleles giving an opportunity to accumulate those alleles and thus achieve crop improvement.

### Conclusions

This study demonstrated that association mapping in elite germplasm seems to have a clear potential for improving sugarcane, especially for complex traits such as CY and SC, for which measurements are costly and time consuming. Combining existing phenotypic trial data and genotypic DArT and TRAP marker characterizations within an LD approach using PCA as a random component to control for population structure may prove to be highly successful to find molecular markers significantly associated with the measured traits. Two aspects were key to obtain the results shown here: the high quality of phenotypic data from the EEAOC-SCBP collected in successive crop cycles and under the same environmental conditions for all genotypes; and the adequate selection of markers to be used in the analysis of population structure, since the choice of markers that do not adequately reflect the presence of such structure could hinder the detection of QTLs of interest. Additionally, sequences of DArT marker associated with trait of interest were aligned in chromosomal regions where sorghum QTLs has been previously reported. The whole role of these regions will need to be further investigated.

Even though the small size of the population could affect the power of the GWAS and increase false positive rate [85], findings reports here must be considered early evidence about the genome regions and markers associated with the genetic control of yield-related characteristics in sugarcane and should be further validated.

## Abbreviations

ASE, allelic substitution effect; CONICET, Consejo Nacional de Investigaciones Científicas y Técnicas; CY, cane yield; DArT, Diversity Arrays Technology; EEAOC, Estación Experimental Agroindustrial Obispo Colombres; GWAS, genome-wide association; IVT, Infield Variety Trials; LD, linkage disequilibrium; MAF, minor allele frequency; MINCYT, Ministerio de Ciencia, Tecnología e Innovación Productiva; PCA, principal component analysis; QTL, quantitative trait loci; SC, sugar content; SCBP-EEAOC, sugarcane breeding program of Estación Experimental Agroindustrial Obispo Colombres; TRAP, target region amplification polymorphism.

## Additional files

Additional file 1: Table S1. Sites, geographic coordinates, and environmental characteristics of the sugarcane trials. (DOCX 13 kb) Additional file 2: Table S2. Dataset of BLUE values obtained for each genotype from model adjusted as described in Methods Section. CY = Cane Yield; SC = Sugar Content. Table S3. Marker dataset for each genotype. (XLSX 520 kb) Additional file 3: Figure S1. Scatterplot matrix and genetic correlation (upper diagonal) between traits. CY = Cane Yield; SC = Sugar Content. (JPG 5656 kb) Additional file 4:
**Table S4**. Dataset of 1745 polymorphic DArT and TRAP markers scored on the 88 sugarcane accessions and used to estimate genetic relationship among clones. **Table S5**. Dice distances matrix. **Table S6**. Ultrametric distances matrix. (XLSX 596 kb) Additional file 5: Figure S2. Quantile-quantile plots for the P-values achieved in the genome-wide association studies (GWAS). (JPG 94 kb) Additional file 6: Table S7. Significant marker-trait associations found in the GWA study on a sugarcane breeding population considering a false-discovery rate (FDR, α = 0.01) to control for multiple testing. Markers associated to more than one trait are in bold. CY = Cane Yield; SC = Sugar Content. (DOCX 26 kb)
