# Supplementary material for: Genome-wide association mapping of quantitative traits in a breeding population of sugarcane
Source: BMC Plant Biol. 2016 Jun 24;16:142. doi: 10.1186/s12870-016-0829-x (PMC4921039; doi:10.1186/s12870-016-0829-x)
Supplement: Additional file 1: Table S1. — Sites, geographic coordinates, and environmental characteristics of the sugarcane trials. (DOCX 13 kb) [file 12870_2016_829_MOESM1_ESM.docx]

Additional file 1. Table S1. Sites, geographic coordinates, and environmental characteristics of the sugarcane trials.

| Sites | Department of Tucumán Province | Average annual rainfall (mm)* | Geographic coordinates (latitude and longitude) | Soil characteristics** | | |
| --- | --- | --- | --- | --- | --- | --- |
|  |  |  |  | Texture | Organic matter | Drainage |
| Cevil Pozo | Cruz Alta | 1178 | 26°84´66´´S  65°12´19´´W | Silt loam | Medium | Moderately well drained |
| Rio Chico | Santa Ana | 1194 | 27°47´41´´S  65°67´55´´W | Loam – Sandy loam | High | Well drained |

*Average of 50 years.

** Classification according the Soil Survey Manual (Soil Survey Staff 1951).
