# Supplementary figures and images for: Genome-wide association mapping of quantitative traits in a breeding population of sugarcane
Source: BMC Plant Biol. 2016 Jun 24;16:142. doi: 10.1186/s12870-016-0829-x (PMC4921039; doi:10.1186/s12870-016-0829-x)

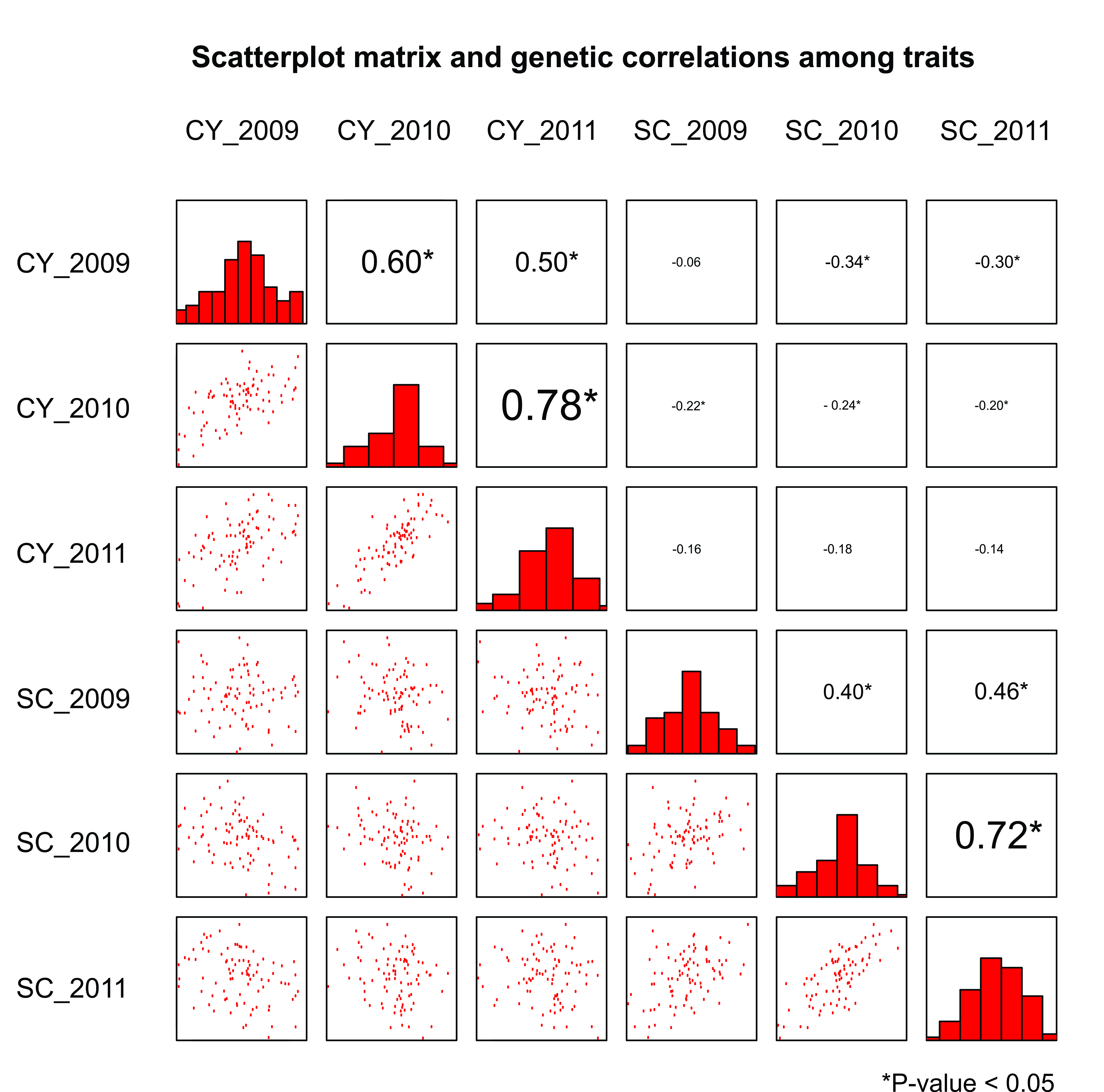

Supplement: Additional file 3: Figure S1. — Scatterplot matrix and genetic correlation (upper diagonal) between traits. CY = Cane Yield; SC = Sugar Content. (JPG 5656 kb) [file 12870_2016_829_MOESM3_ESM.jpg]

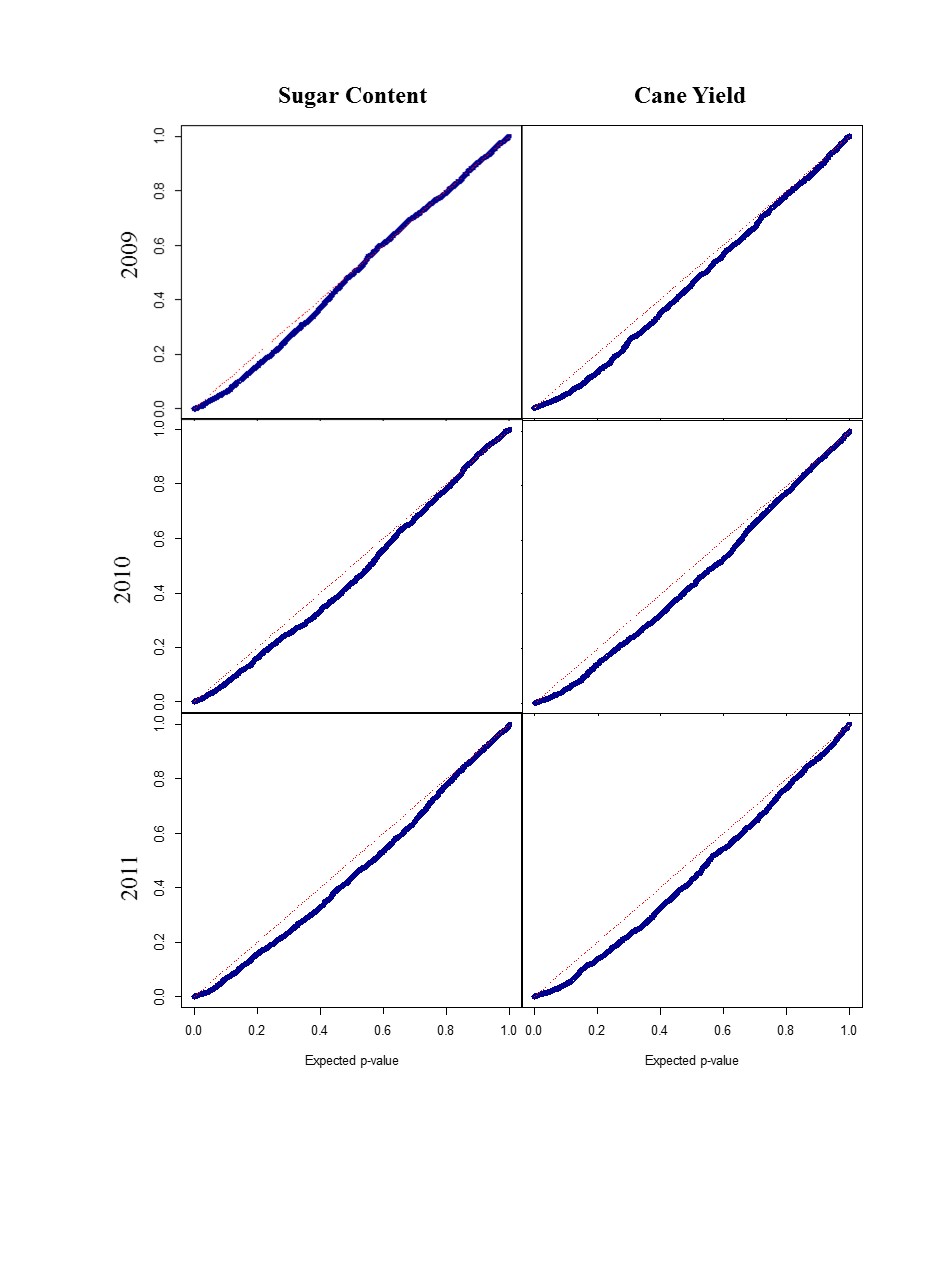

Supplement: Additional file 5: Figure S2. — Quantile-quantile plots for the P-values achieved in the genome-wide association studies (GWAS). (JPG 94 kb) [file 12870_2016_829_MOESM5_ESM.jpg]
