# Supplementary material for: Genome-wide association mapping of quantitative traits in a breeding population of sugarcane
Source: BMC Plant Biol. 2016 Jun 24;16:142. doi: 10.1186/s12870-016-0829-x (PMC4921039; doi:10.1186/s12870-016-0829-x)
Supplement: Additional file 6: Table S7. — Significant marker-trait associations found in the GWA study on a sugarcane breeding population considering a false-discovery rate (FDR, α = 0.01) to control for multiple testing. Markers associated to more than one trait are in bold. CY = Cane Yield; SC = Sugar Content. (DOCX 26 kb) [file 12870_2016_829_MOESM6_ESM.docx]

**Additional file 6.** Table S7. Significant marker-trait associations found in the GWA study on a sugarcane breeding population considering a false-discovery rate (FDR, α=0.01) to control for multiple testing. Markers associated to more than one trait are in bold. CY=Cane Yield; SC=Sugar Content.

| locus | p.val | Trait |
| --- | --- | --- |
| M1 | 0.0025 | SC_2010 |
| M10 | 0.00369 | CY_2010 |
| M100 | 0.0083 | CY_2011 |
| M101 | 0.000394 | SC_2011 |
| M102 | 0.00607 | SC_2009 |
| M103 | 0.003 | SC_2010 |
| M104 | 0.00534 | CY_2009 |
| M105 | 0.006 | CY_2011 |
| M106 | 0.0046 | SC_2010 |
| M107 | 0.00465 | CY_2010 |
| **M108** | **0.00868** | CY_2009 |
| **M108** | **0.00997** | CY_2011 |
| M109 | 0.00664 | CY_2011 |
| M11 | 0.00666 | CY_2010 |
| M110 | 0.0051 | SC_2010 |
| M111 | 0.00891 | SC_2009 |
| M112 | 0.00126 | CY_2009 |
| M113 | 0.00725 | SC_2009 |
| M114 | 0.00314 | CY_2009 |
| M115 | 0.007293 | SC_2011 |
| M116 | 0.00125 | SC_2009 |
| M117 | 0.00885 | CY_2009 |
| M118 | 0.00361 | CY_2010 |
| M119 | 0.0021 | CY_2009 |
| M12 | 0.0094 | SC_2010 |
| **M120** | **0.00304** | CY_2010 |
| **M120** | **0.00547** | CY_2011 |
| M121 | 0.00755 | CY_2010 |
| M122 | 0.009293 | SC_2011 |
| M123 | 0.0021 | CY_2009 |
| M124 | 0.0027 | SC_2010 |
| **M125** | **0.00504** | CY_2010 |
| **M125** | **0.00383** | CY_2011 |
| M126 | 0.00897 | CY_2009 |
| M127 | 0.00893 | CY_2010 |
| M128 | 0.00885 | CY_2009 |
| M129 | 0.0066 | SC_2010 |
| M13 | 0.00886 | SC_2009 |
| M130 | 0.00489 | CY_2009 |
| M131 | 0.00629 | CY_2011 |
| M132 | 0.00041 | SC_2009 |
| M133 | 0.00837 | SC_2009 |
| M134 | 0.00981 | CY_2010 |
| M135 | 0.003598 | SC_2011 |
| M136 | 0.00455 | CY_2011 |
| M137 | 0.0051 | SC_2010 |
| M138 | 0.00629 | CY_2011 |
| M139 | 0.00302 | SC_2011 |
| **M14** | **0.0027** | SC_2010 |
| **M14** | **0.007375** | SC_2011 |
| M140 | 0.00926 | CY_2010 |
| M141 | 0.00551 | CY_2009 |
| M142 | 0.0072 | SC_2010 |
| M143 | 0.00704 | CY_2010 |
| M144 | 0.00052 | SC_2009 |
| M145 | 0.0027 | CY_2011 |
| M146 | 0.00704 | CY_2010 |
| M147 | 0.00313 | SC_2009 |
| M148 | 0.00175 | CY_2009 |
| M149 | 0.00878 | CY_2011 |
| M15 | 0.00486 | SC_2011 |
| M150 | 0.002794 | SC_2011 |
| **M151** | **0.00126** | CY_2009 |
| **M151** | **0.00285** | CY_2011 |
| M152 | 0.00096 | SC_2009 |
| **M153** | **0.0097** | SC_2010 |
| **M153** | **0.001634** | SC_2011 |
| M154 | 0.00175 | CY_2009 |
| M155 | 0.00988 | CY_2009 |
| M156 | 0.00112 | SC_2009 |
| M157 | 0.00887 | SC_2009 |
| M158 | 0.00613 | SC_2009 |
| M159 | 0.008542 | SC_2011 |
| M16 | 0.00369 | CY_2009 |
| M160 | 0.003248 | SC_2011 |
| M161 | 0.005429 | SC_2011 |
| M162 | 0.001456 | SC_2011 |
| M163 | 0.00818 | CY_2010 |
| M164 | 0.00436 | SC_2009 |
| M165 | 0.0088 | CY_2011 |
| M166 | 0.00776 | CY_2011 |
| M167 | 0.00334 | CY_2011 |
| **M168** | **0.00667** | SC_2009 |
| **M168** | **0.0016** | SC_2010 |
| M169 | 0.00595 | CY_2009 |
| M17 | 0.00383 | CY_2009 |
| M170 | 0.0067 | SC_2010 |
| M171 | 0.0085 | SC_2010 |
| M172 | 0.0059 | SC_2010 |
| **M173** | **0.00665** | CY_2010 |
| **M173** | **0.009** | SC_2010 |
| **M173** | **0.009658** | SC_2011 |
| M174 | 0.00269 | CY_2011 |
| M175 | 0.004562 | SC_2011 |
| M176 | 0.00194 | SC_2009 |
| M177 | 0.003 | SC_2010 |
| M178 | 0.00175 | CY_2009 |
| M179 | 0.0084 | CY_2009 |
| M18 | 0.00173 | CY_2010 |
| M180 | 0.00309 | CY_2009 |
| **M181** | **0.00052** | SC_2009 |
| **M181** | **0.004116** | SC_2011 |
| M182 | 0.00926 | CY_2009 |
| M183 | 0.0089 | SC_2010 |
| M184 | 0.0043 | SC_2010 |
| M185 | 0.00041 | CY_2009 |
| M186 | 0.0069 | SC_2010 |
| M187 | 0.00902 | CY_2010 |
| **M188** | **0.00428** | CY_2010 |
| **M188** | **0.0085** | SC_2010 |
| **M188** | **0.007623** | SC_2011 |
| **M189** | **0.00808** | CY_2010 |
| **M189** | **0.00552** | CY_2011 |
| M19 | 0.006378 | SC_2011 |
| M190 | 0.0018 | SC_2010 |
| M191 | 0.00306 | CY_2010 |
| M192 | 0.002037 | SC_2011 |
| **M193** | **0.00872** | CY_2010 |
| **M193** | **0.00044** | CY_2011 |
| M194 | 0.009164 | SC_2011 |
| M195 | 0.00499 | CY_2009 |
| M196 | 0.007134 | SC_2011 |
| **M197** | **0.00047** | CY_2009 |
| **M197** | **0.00287** | CY_2010 |
| **M198** | **0.00265** | CY_2009 |
| **M198** | **0.00487** | CY_2011 |
| M199 | 0.00103 | SC_2009 |
| M2 | 0.00683 | CY_2009 |
| M20 | 0.00405 | CY_2011 |
| M200 | 0.0001 | CY_2009 |
| M201 | 0.00808 | CY_2011 |
| M202 | 0.0001 | CY_2011 |
| **M203** | **0.00938** | CY_2009 |
| **M203** | **0.0088** | CY_2010 |
| M204 | 0.00101 | CY_2009 |
| M205 | 0.006197 | SC_2011 |
| M206 | 0.0043 | SC_2010 |
| M207 | 0.0089 | CY_2009 |
| M21 | 0.00456 | CY_2010 |
| M22 | 0.007098 | SC_2011 |
| M23 | 0.00164 | CY_2011 |
| M24 | 0.00138 | CY_2010 |
| M25 | 0.00426 | CY_2011 |
| M26 | 0.00122 | SC_2009 |
| M27 | 0.00767 | CY_2011 |
| M28 | 0.00017 | SC_2009 |
| M29 | 0.00662 | CY_2010 |
| M3 | 0.00886 | SC_2009 |
| **M30** | **0.00702** | CY_2009 |
| **M30** | **0.00085** | CY_2010 |
| M31 | 0.00303 | SC_2009 |
| **M32** | **0.00276** | CY_2010 |
| **M32** | **0.00267** | CY_2011 |
| M33 | 0.001791 | SC_2011 |
| M34 | 0.002124 | SC_2011 |
| M35 | 0.00557 | CY_2009 |
| M36 | 0.007532 | SC_2011 |
| M37 | 0.008316 | SC_2011 |
| M38 | 0.00572 | CY_2010 |
| M39 | 0.008923 | SC_2011 |
| M4 | 0.0046 | SC_2010 |
| M40 | 0.00048 | SC_2009 |
| **M41** | **0.0089** | SC_2010 |
| **M41** | **0.000798** | SC_2011 |
| M42 | 0.00438 | CY_2011 |
| M43 | 0.00858 | SC_2009 |
| M44 | 0.00768 | SC_2009 |
| **M45** | **0.00045** | SC_2009 |
| **M45** | **0.002219** | SC_2011 |
| **M46** | **0.00478** | CY_2009 |
| **M46** | **0.00266** | CY_2010 |
| M47 | 0.00728 | CY_2011 |
| M48 | 0.00232 | CY_2011 |
| M49 | 0.00848 | SC_2009 |
| **M5** | **0.0029** | SC_2010 |
| **M5** | **0.007517** | SC_2011 |
| **M50** | **0.00196** | CY_2010 |
| **M50** | **0.00267** | CY_2011 |
| M51 | 0.009005 | SC_2011 |
| M52 | 0.00141 | CY_2009 |
| M53 | 0.0077 | CY_2011 |
| **M54** | **0.00024** | CY_2010 |
| **M54** | **0.00156** | CY_2011 |
| **M54** | **0.001503** | SC_2011 |
| M55 | 0.00527 | CY_2009 |
| M56 | 0.00218 | CY_2010 |
| **M57** | **0.0085** | SC_2010 |
| **M57** | **0.000081** | SC_2011 |
| **M58** | **0.00021** | CY_2010 |
| **M58** | **0.00155** | CY_2011 |
| **M58** | **0.002452** | SC_2011 |
| **M59** | **0.00171** | CY_2010 |
| **M59** | **0.00255** | CY_2011 |
| M6 | 0.00436 | CY_2010 |
| M60 | 0.00444 | CY_2011 |
| M61 | 0.00515 | CY_2009 |
| M62 | 0.00347 | CY_2010 |
| M63 | 0.00383 | SC_2009 |
| **M64** | **0.00105** | SC_2009 |
| **M64** | **0.0058** | SC_2010 |
| **M64** | **0.007222** | SC_2011 |
| M65 | 0.0088 | SC_2010 |
| M66 | 0.0031 | CY_2009 |
| M67 | 0.0081 | CY_2009 |
| M68 | 0.00848 | CY_2011 |
| M69 | 0.00639 | CY_2009 |
| M7 | 0.00209 | CY_2010 |
| M70 | 0.0062 | SC_2010 |
| **M71** | **0.00163** | CY_2010 |
| **M71** | **0.00695** | CY_2011 |
| M72 | 0.00305 | CY_2010 |
| M73 | 0.004341 | SC_2011 |
| M74 | 0.00235 | SC_2009 |
| M75 | 0.00994 | SC_2009 |
| M76 | 0.003327 | SC_2011 |
| M77 | 0.003472 | SC_2011 |
| **M78** | **0.00292** | SC_2009 |
| **M78** | **0.004627** | SC_2011 |
| M79 | 0.00607 | CY_2009 |
| M8 | 0.00117 | CY_2009 |
| M80 | 0.0019 | SC_2010 |
| M81 | 0.00029 | SC_2009 |
| M82 | 0.009524 | SC_2011 |
| M83 | 0.0031 | SC_2010 |
| M84 | 0.007042 | SC_2011 |
| M85 | 0.00023 | SC_2009 |
| **M86** | **0.0014** | SC_2010 |
| **M86** | **0.007212** | SC_2011 |
| M87 | 0.00064 | SC_2009 |
| M88 | 0.002688 | SC_2011 |
| M89 | 0.00164 | SC_2009 |
| M9 | 0.0049 | SC_2010 |
| M90 | 0.0098 | SC_2011 |
| **M91** | **0.00751** | CY_2009 |
| **M91** | **0.00294** | CY_2010 |
| M92 | 0.009231 | SC_2011 |
| M93 | 0.00145 | SC_2009 |
| M94 | 0.00932 | SC_2009 |
| **M95** | **0.00185** | CY_2010 |
| **M95** | **0.0022** | CY_2011 |
| M96 | 0.005692 | SC_2011 |
| **M97** | **0.00467** | CY_2010 |
| **M97** | **0.0011** | CY_2011 |
| M98 | 0.00145 | CY_2011 |
| M99 | 0.00706 | CY_2011 |
